# Association of serum selenium with MASLD and liver fibrosis: A cross-sectional study

**DOI:** 10.1371/journal.pone.0314780

**Published:** 2024-12-31

**Authors:** Wenying Guo, Ting Weng, Yufei Song

**Affiliations:** Ningbo Medical Center Lihuili Hospital of Ningbo University, Ningbo, Zhejiang, People’s Republic of China; Universita degli Studi della Campania Luigi Vanvitelli Scuola di Medicina e Chirurgia, ITALY

## Abstract

**Background:**

The evolution of NAFLD, MAFLD, and MASLD underscores significant advancements and nomenclatural shifts in the realm of chronic liver disorders. This study primarily aimed to investigate the possible link between serum selenium levels and the occurrence of MASLD.

**Methods:**

Utilizing data from NHANES for the years 2017 through 2020, we performed an in-depth analysis. To evaluate the relationship between serum selenium concentrations and the prevalence of MASLD and liver fibrosis, we employed a comprehensive multivariable analysis. This approach accounted for a range of variables to enhance the robustness and reliability of our results by mitigating potential confounding factors.

**Results:**

Through the application of linear regression models, our comprehensive data analysis revealed significant insights. Elevated serum selenium levels exhibited a distinct positive correlation with CAP, whereas an inverse relationship with LSM was observed. Multivariate logistic regression analysis indicated that higher serum selenium concentrations were significantly associated with an increased likelihood of MASLD, alongside a marked reduction in the probability of liver fibrosis.

**Conclusion:**

The findings of this study highlight a significant positive association between elevated serum selenium levels, CAP, and the prevalence of MASLD, coupled with an inverse relationship with LSM and liver fibrosis.

## 1. Introduction

Non-Alcoholic Fatty Liver Disease (NAFLD), first described in the 1980s, refers to the pathological accumulation of fat in the liver in the absence of significant alcohol intake [1]. The term encompasses a spectrum of hepatic conditions, ranging from simple steatosis to non-alcoholic steatohepatitis (NASH), which may further progress to fibrosis and cirrhosis [1]. However, the term NAFLD has faced criticism for lacking specificity and failing to reflect the complex etiology of the disease, creating challenges for accurate diagnosis and research [2]. As a result, the term metabolic-associated fatty liver disease (MAFLD) was introduced to better capture the underlying metabolic dysfunctions [2]. MAFLD highlights the metabolic basis of fatty liver disease and includes diagnostic criteria encompassing metabolic syndrome features like obesity, type 2 diabetes (T2DM), and other metabolic abnormalities. This change aims to offer a more precise and inclusive disease definition, improving patient management and therapeutic strategies [3]. Recently, the term metabolic-associated steatosis liver disease (MASLD) has emerged, refining the classification of liver diseases by emphasizing metabolic factors contributing to liver steatosis [4]. MASLD highlights the crucial role of metabolic dysregulation in liver disease pathogenesis, promoting a targeted approach for diagnosis and treatment [4].

Selenium (Se) is a vital trace mineral necessary for human health maintenance [5]. In the human body, selenium predominantly exists as selenocysteine (SeCys), which is essential for various biological processes. The human selenoproteome consists of 25 selenoproteins that possess antioxidant properties. These proteins are crucial in modulating reactive oxygen species (ROS) production, controlling apoptosis, managing endoplasmic reticulum stress, and regulating diverse pathophysiological processes [6].

Previous research has examined the impact of serum selenium levels on MASLD and liver fibrosis [7–9]. However, the association between serum selenium concentrations and MASLD using Vibration-Controlled Transient Elastography (VCTE) has not been investigated. Furthermore, prior studies have typically analyzed serum selenium and liver fibrosis using data from a single cycle, specifically 2017–2018, which limits the sample size and representativeness of the studied population. This study aims to use the Controlled Attenuation Parameter (CAP) and Liver Stiffness Measurement (LSM) derived from VCTE data in the National Health and Nutrition Examination Survey (NHANES) 2017–2020 cycle. The objective of this study is to explore the cross-sectional relationship between serum selenium levels and both MASLD and liver fibrosis.

## 2. Materials and methods

### 2.1 Study cohort

**Fig 1:**
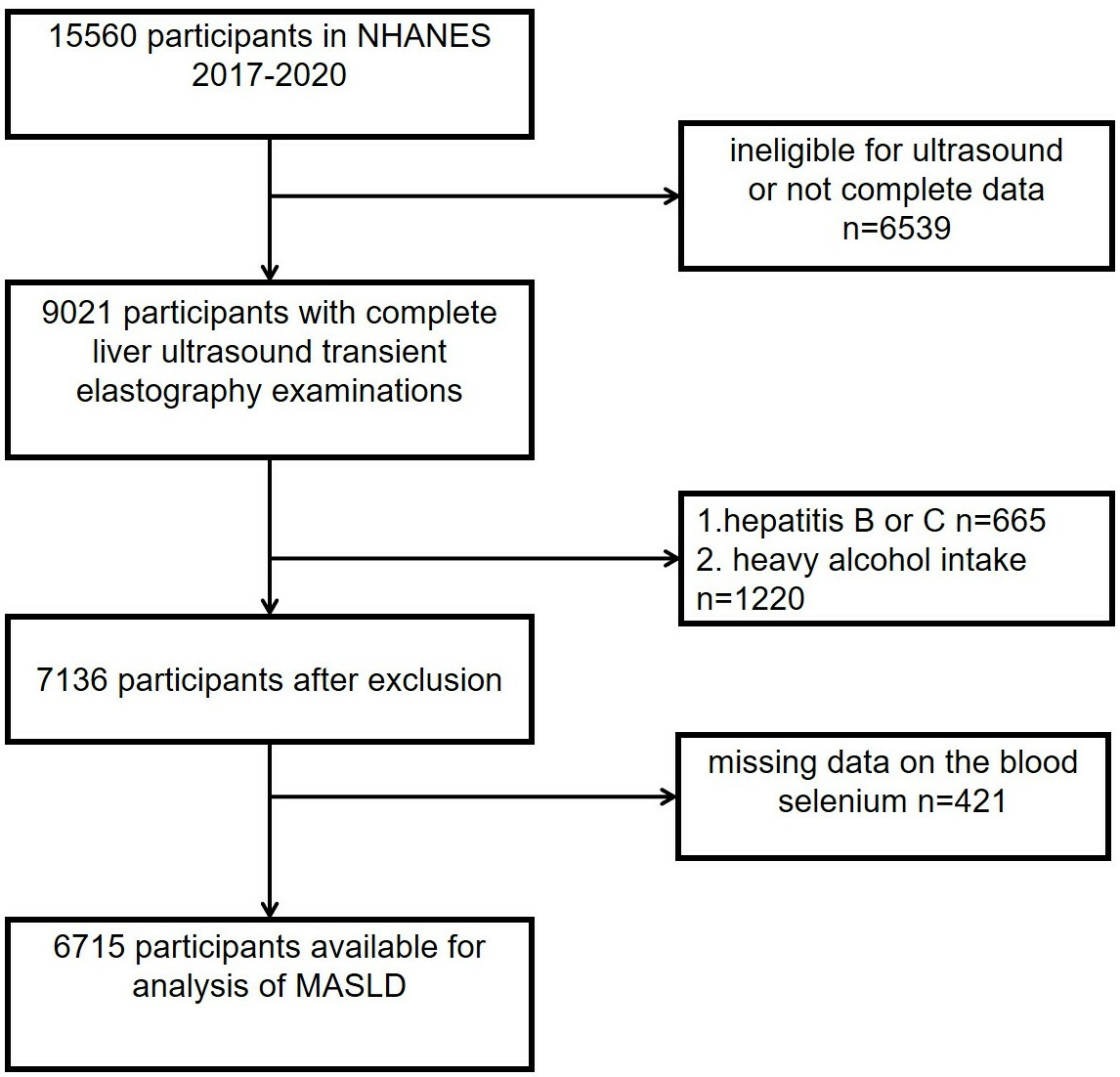
Flow chart of participants screening.

The study was supported by the National Centre for Health Statistics Research Ethics Review Board. The dataset utilized in this research was sourced from the NHANES cycle covering the years 2017 to 2020. The initial sample consisted of 15,560 individuals, with exclusions made for the following reasons: (1) incomplete liver elastography data (n = 6,539), (2) diagnosis of viral hepatitis (n = 665), (3) excessive alcohol consumption, defined as ≥3 drinks per day for males and ≥2 drinks per day for females (n = 1,220), and (4) missing serum selenium data (n = 421). Following these exclusions, the final analytical sample included 6,715 participants. A comprehensive overview is provided in Fig 1.

### 2.2 Serum selenium quantification

During the research, serum specimens were gathered, preserved at -30°C, and forwarded to the CDC in Atlanta, Georgia, for further examination. The quantification of selenium in the serum was performed using inductively coupled plasma dynamic reaction cell mass spectrometry, in compliance with NHANES quality assurance and quality control guidelines. The lower limit of detection (LLOD) for selenium was determined to be 24.48 μg/L, and the measured serum selenium concentrations in this study surpassed this LLOD.

### 2.3 The definition of MASLD

The diagnosis of MASLD was established in the presence of hepatic steatosis and the absence of significant alcohol consumption or viral hepatitis. The following criteria were taken into account: (1) Individuals with a body mass index (BMI) ≥ 25 kg/m² or a waist circumference (WC) ≥ 94 cm for males and ≥ 80 cm for females. (2) Fasting plasma glucose (FPG) levels ≥ 100 mg/dL or hemoglobin A1c (Hb A1c) level ≥ 5.7%, or a previous diagnosis of T2DM or current treatment for T2DM. (3) Serum pressure levels ≥ 130/85 mmHg or current treatment for hypertension. (4) Individuals with triglyceride (TG) levels ≥ 1.70 mmol/L or currently undergoing lipid-lowering therapy. (5) Low levels of high-density lipoprotein cholesterol (HDL) < 1.0 mmol/L for males or < 1.3 mmol/L for females, or those currently undergoing lipid-lowering therapy [4].

### 2.4 Non-invasive liver steatosis and fibrosis assessment

This study implemented VCTE for the quantification of hepatic steatosis. Participants were subjected to a fasting period of no less than three hours and tasked with obtaining more than ten liver measurements, aiming to achieve an interquartile range/median ratio below 30% to ensure measurement accuracy. In line with established literature, hepatic steatosis was diagnosed by CAP score equal to or exceeding 248 dB/m [10, 11], while liver fibrosis was identified by LSM score equal to or exceeding 7 kPa [7, 12]. Additionally, the hepatic steatosis index (HSI) and fibrosis-4 index (FIB-4) were utilized to assess hepatic steatosis and fibrosis, respectively [13, 14].

### 2.5 Covariate evaluation

The laboratory conducted measurements on FPG, WC, TG, total cholesterol (TC), low-density lipoprotein cholesterol (LDL), HDL and HbA1c. Demographic data including age, sex, ethnicity, educational attainment, marital status, poverty income ratio (PIR), alcohol consumption, and medication usage were collected via standardized self-reported questionnaires. Physical activity (PA) was assessed through the calculation PA = MET (Metabolic Equivalent) × frequency per week × duration of each activity. PA = 0 signified absence of physical activity. Participants were classified based on stipulate reaching a minimum of 600 MET-minutes per week for adults [15]. Diabetes was determined based on FPG levels equal to or greater than 7 mmol/L, HbA1c levels equal to or greater than 6.5%, self-reported clinician-diagnosed diabetes, or the use of diabetes medication [16]. Hypertension was diagnosed using criteria such as having a serum pressure surpassing 130/80 mm Hg or the utilization of antihypertensive medications [17]. Participants were segregated into two cohorts based on their alcohol intake: those who abstained from alcohol and those who consumed alcohol in moderation (1–2 drinks per day for males, 1 drink per day for females). Smoking status was evaluated by assessing cotinine levels [18, 19]. The PIR was categorized into three groups: <1.30, 1.30–3.50, and≥3.50 [20].

### 2.6 Statistical methodology

Continuous parameters were denoted by their mean values accompanied by standard deviations, whereas categorical factors were exhibited in percentages. A weighted t-test was applied to juxtapose continuous variables, and a chi-squared examination was employed for assessing categorical factors. Given the low proportion of missing values in the dataset, we employed repetitive imputation to manage the missing data. The investigation regarding the correlation between serum selenium concentrations with CAP and LSM leveraged a linear regression model. Three distinct analytical frameworks were constructed to probe the intricate relationship between covariates and the resultant analytical outcomes, with each model progressively incorporating nuanced adjustments. The foundational model remained unaltered, whereas the subsequent model introduced tailored modifications for specific variables including PIR, PA, BMI, age, ethnicity, gender, educational attainment, and marital status. The comprehensively adjusted third model additionally encompassed supplementary factors such as smoking habits, alcohol consumption, diabetes prevalence, and hypertension. The correlation between serum selenium levels in MASLD and liver fibrosis was assessed using a multivariable logistic regression model, as previously described. Subgroup analyses were conducted to examine variations in effect measures, considering gender (male/female), age (<60, ≥60), BMI (<28, ≥28), diabetes, and hypertension as potential influencing factors. Additionally, restricted cubic spline (RCS) analysis was employed to investigate potential nonlinear relationships between serum selenium levels in MASLD and liver fibrosis. A significance threshold of p < 0.05 was applied to all statistical analyses, with R (version 4.1.0, Vienna, Austria) utilized as the analytical software tool.

## 3. Results

### 3.1 Participant baseline characteristic

In our initial examination, we included a total of 6,715 participants, classifying them into four quartiles based on their serum selenium concentrations. Additional baseline characteristics for these participants are detailed in S1 Table. We observed substantial differences across the quartiles in relation to gender, race, marital status, alcohol consumption, smoking habits, and the prevalence of MASLD and liver fibrosis. Importantly, as serum selenium levels increased, there was a corresponding rise in age, waist circumference, TC, TG, FPG, LDL, HbA1c, and CAP.

### 3.2 Correlation between serum selenium and CAP, LSM

The results detailed in Table 1 offer insights from a series of multiple linear regression analyses conducted to explore the potential relationship between serum selenium levels and both CAP and LSM. In the first model (Model 1), a significant positive association was observed between serum selenium and CAP, while a significant negative association was found between serum selenium and LSM (p = 0.043 and 0.035, respectively). In the following phase, Model 2 was presented, and intriguingly, this modified version continued to display a significant relationship between serum selenium concentrations and both CAP and LSM (p = 0.047 and 0.011, respectively). Ultimately, Model 3 consistently revealed a positive linkage between serum selenium and CAP, while a negative association remained evident between serum selenium and LSM (p = 0.011 and 0.006, respectively).

**Table 1:** Linear regression analysis of serum selenium with CAP and LSM.

**CAP**	**model1**	**β, (95% CI)**	**0.178(0.093,0.263)**
		P-value	0.043
	model2	β, (95% CI)	0.079(0.002,0.157)
		P-value	0.047
	model3	β, (95% CI)	0.102(0.023,0.180)
		P-value	0.011
**LSM**	model1	β, (95% CI)	-0.003(-0.007,-0.003)
		P-value	0.035
	model2	β, (95% CI)	-0.005(-0.009,-0.001)
		P-value	0.011
	model3	β, (95% CI)	-0.005(-0.009,-0.002)
		P-value	0.006

### 3.3 Correlation between serum selenium and MASLD, liver fibrosis

Table 2 displays the findings from multiple logistic regression analyses examining the independent associations between serum selenium levels, MASLD, and liver fibrosis. In Model 1, a significant positive relationship was found between higher serum selenium levels (Q3, Q4) and MASLD (both P<0.05), while a significant negative relationship was noted between the highest serum selenium levels and liver fibrosis (P = 0.035). Model 2 further validated these positive associations, demonstrating significant links between MASLD and serum selenium levels (Q3, Q4, P = 0.005 and 0.004, respectively), and confirmed the significant negative association for the highest serum selenium levels with liver fibrosis (P = 0.046). The final model, Model 3, continued to show a significant increase in the likelihood of MASLD with elevated serum selenium levels (Q3, Q4, P = 0.004 and 0.008, respectively), while the significant negative association with liver fibrosis at the highest serum selenium levels remained (P = 0.027). S2 Table presents the findings for liver steatosis and fibrosis, as evaluated by hepatic steatosis index (HSI) and fibrosis index based on the 4 factors (FIB-4). A significant reduction in the incidence of liver fibrosis, as measured by FIB-4, was observed with higher serum selenium levels. In contrast, no significant association was found between MASLD, as defined by HSI, and serum selenium levels.

**Table 2:** Logistic regression analysis of serum selenium and MASLD, Liver Fibrosis diagnosed by VCTE.

			Q1	Q2	Q3	Q4
**MASLD**	model1	OR (95%CI)	ref	0.982(0.798,1.208)	1.382(1.121,1.705)	1.582(1.279,1.957)
		P trend	ref	0.861	0.002	0.001
	model2	OR (95%CI)	ref	0.962(0.748,1.239)	1.419(1.110,1.814)	1.470(1.129,1.914)
		P trend	ref	0.767	0.005	0.004
	model3	OR (95%CI)	ref	0.956(0.744,1.228)	1.431(1.118,1.830)	1.432(1.097,1.868)
		P trend	ref	0.723	0.004	0.008
**Liver fibrosis**	model1	OR (95%CI)	ref	0.820(0.612,1.098)	0.777(0.566,1.066)	0.597(0.370,0.965)
		P trend	ref	0.183	0.118	0.035
	model2	OR (95%CI)	ref	0.803(0.594,1.086)	0.741(0.537,1.021)	0.739(0.549,0.995)
		P trend	ref	0.154	0.067	0.046
	model3	OR (95%CI)	ref	0.804(0.593,1.090)	0.743(0.539,1.023)	0.709(0.522,0.962)
		P trend	ref	0.161	0.069	0.027

**Fig 2:**
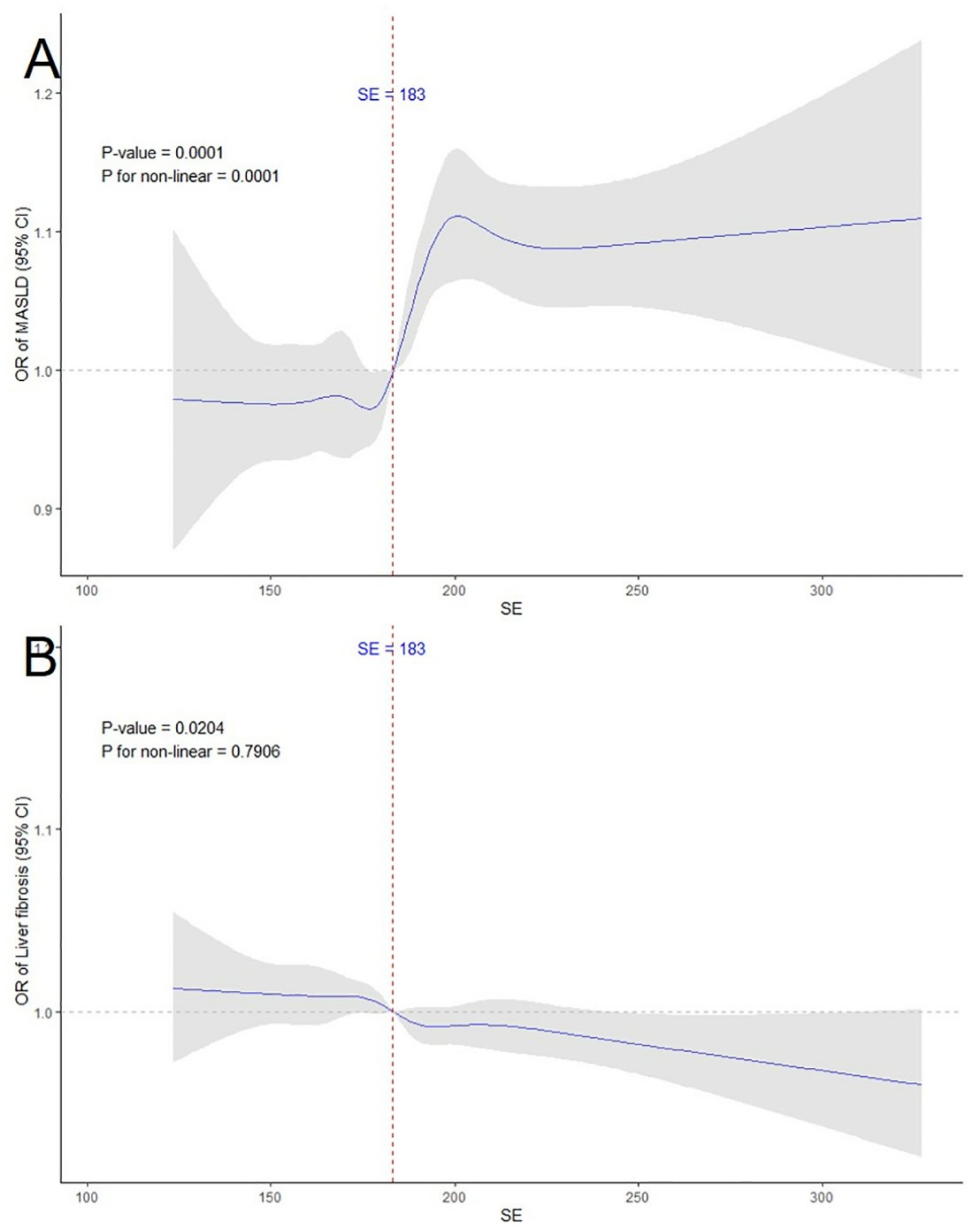
RCS plots of the association of selenium with (A) MASLD, (B) liver fibrosis.

After extensive multivariable adjustments in Model 3, a significant and nonlinear relationship was identified between serum selenium levels and the occurrence of MASLD through RCS analysis (p overall = 0.0001; p nonlinear = 0.0001). Conversely, no significant nonlinear relationship was found between serum selenium and liver fibrosis (p overall = 0.0002; p nonlinear = 0.7906), as illustrated in Fig 2.

### 3.4 Subgroup analysis

**Fig 3:**
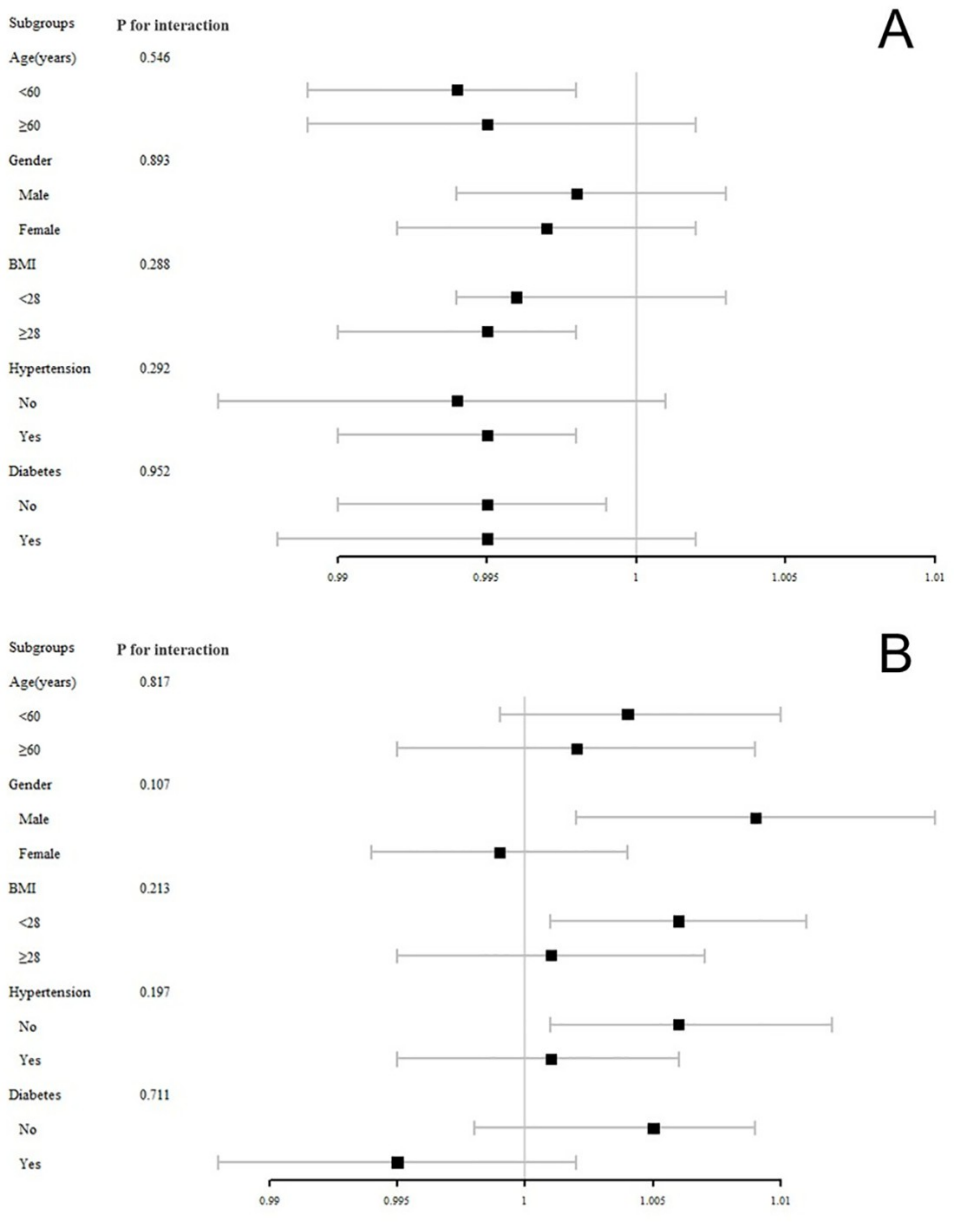
Subgroup analysis of the association between selenium with (A) MASLD, (B) liver fibrosis.

To further explore the association between serum selenium and the incidence of MASLD and liver fibrosis, we conducted stratified multivariate regression analyses across various population subgroups based on gender, age, BMI, diabetes, and hypertension. The findings, depicted in Fig 3, indicate that none of the subgroup analyses revealed statistically significant associations for either MASLD or liver fibrosis (p all > 0.05). Additionally, we conducted propensity score matching followed by logistic regression analyses to explore the associations between serum selenium levels, MASLD, and liver fibrosis. The results, as presented in S3 and S4 Tables, further validate the robustness of our findings.

## 4. Discussion

Previous research has primarily focused on exploring the association between serum selenium concentrations and the risk of MASLD, as well as liver fibrosis [7–9, 21]. However, there remains a notable lack of epidemiological data on the potential association between serum selenium and MASLD as measured by VCTE. In addition, studies using VCTE to assess the correlation between serum selenium levels and liver fibrosis have relatively small sample sizes [7, 8]. To address this gap, we analyzed NHANES survey data from the 2017–2020 cycles. Our study found a significant positive correlation between serum selenium levels and MASLD, and a negative correlation with liver fibrosis. These findings align with prior research and remain robust after adjusting for multiple potential confounders [7–9, 21]. However, our subgroup analyses did not show any significant variable differences.

Historically, selenium has been recognized for its important role in metabolic syndrome and its potential to slow the progression of NAFLD [22–24]. Selenium can enhance the expression of enzymes involved in lipid oxidation while inhibiting those responsible for de novo lipogenesis, thus mitigating steatosis through dual mechanisms [25]. Additionally, selenium strengthens the liver’s antioxidant capacity by increasing glutathione peroxidase activity [26]. This boost in antioxidant capacity is crucial, as oxidative stress plays a major role in liver cell injury and steatosis. By upregulating glutathione peroxidase, selenium neutralizes ROS, reducing cellular oxidative damage—a known contributor to NAFLD progression [25]. Selenium shows significant inhibitory effects on metalloproteinases, tumor necrosis factor-alpha (TNF-α), interleukin-6 (IL-6), transforming growth factor-beta1, and other cytokines and growth factors involved in NAFLD development, helping to reduce hepatic inflammation [27, 28]. These cytokines play a central role in the inflammatory cascade that worsens liver injury and fibrosis. For example, TNF-α and IL-6 activate inflammatory signaling pathways like nuclear transcription factor kappaB (NF-κB), resulting in increased levels of pro-inflammatory mediators. Selenium’s suppression of these pathways may be a key mechanism by which it reduces liver inflammation and slows fibrosis progression [27, 28].

Previous epidemiological studies have shown a strong inverse relationship between serum selenium levels and liver fibrosis incidence, a result further confirmed by larger-scale studies [7, 8]. In contrast, other studies have found a positive association between higher selenium exposure and MASLD, consistent with our findings [9]. This paradoxical role of selenium, which may both alleviate and worsen liver disease, could be dose-dependent. At physiological levels, selenium supports antioxidant and anti-inflammatory pathways. However, at elevated levels, it may cause harmful oxidative stress and inflammatory responses by over activating the NF-κB and MAPK pathways. This dual action illustrates the complex role of selenium in liver pathology [29, 30]. Furthermore, selenium can induce oxidative stress and liver damage. Animal studies have shown that excessive selenium intake may lead to liver lipid accumulation [31]. Elevated selenium levels may upregulate selenoproteins and glutathione peroxidase, leading to excess hydrogen peroxide production. Excessive hydrogen peroxide can overwhelm the cell’s antioxidant defenses. Selenium-induced oxidative stress may worsen lipid peroxidation and mitochondrial dysfunction, further damaging liver cells and disrupting metabolism. Such oxidative stress decreases the phosphorylation of the insulin receptor subunit and Akt, reducing insulin sensitivity and increasing lipid peroxidation [32]. Therefore, further research is needed to clarify the relationship between serum selenium levels, MASLD, and liver fibrosis.

Subgroup analysis did not identify a significant correlation between serum selenium levels and MASLD or liver fibrosis. This finding aligns with previous research, which suggests minimal variation in serum selenium levels relative to age in NAFLD [8, 33]. Moreover, previous studies have consistently demonstrated a positive correlation between serum selenium levels and liver steatosis in males [7]. However, this study found no positive correlation between serum selenium levels and MASLD after gender adjustment, possibly due to different diagnostic criteria for MASLD. In an age subgroup analysis of liver fibrosis, Liu et al. found that serum selenium levels in elderly participants had a more pronounced protective effect against liver fibrosis compared to younger participants. This observation, not present in our study, may be due to the significant difference in sample sizes between the two subgroups. Previous studies have shown that the protective effect of serum selenium against liver fibrosis is less pronounced in women than in men. Studies have indicated that serum selenium concentrations are typically lower in women than in men [34]. This disparity may be due to the greater vulnerability of the female liver to conditions like drug-induced liver damage and dysfunction compared to males [35]. Therefore, further investigation is needed, especially in the context of the MASLD study, to clarify the mechanisms by which selenium levels affect liver fibrosis.

To our knowledge, this is the first study to investigate the relationship between serum selenium levels and MASLD using VCTE. We also explored the relationship between serum selenium levels and liver fibrosis using extensive VCTE data. However, several limitations should be noted. First, the cross-sectional nature of the NHANES dataset limits our ability to infer causality, as it captures data at only one point in time. This prevents us from establishing temporal sequences or determining cause-and-effect relationships, which should be considered a limitation in interpreting the findings. Additionally, some lifestyle factors, such as alcohol intake and PA, were primarily self-reported, potentially introducing bias. Although multiple covariates have been adjusted for, confounding factors may still affect the observed associations between serum selenium, MASLD, and liver fibrosis. Previous research has identified a significant inverse correlation between MASLD incidence and an increased combined dietary antioxidant index, which incorporates various vitamins and trace elements [36]. The potential impact of biologically active supplements, particularly selenium, on these findings must be considered. The NHANES dataset includes self-reported data on dietary supplement use but does not distinguish between natural dietary selenium intake and that from supplements. This limitation complicates the isolation of selenium’s true effect from dietary sources alone, suggesting that dietary supplements may confound our analysis. The most definitive method for diagnosing hepatic steatosis and fibrosis is liver biopsy, which is unavailable in NHANES. While VCTE data can be used, ongoing debate surrounds the specific cutoffs for different stages.

## 5. Conclusion

This comprehensive cross-sectional study, involving a large cohort, revealed a significant positive correlation between elevated serum selenium levels and CAP, which suggests a higher degree of liver steatosis. In contrast, a negative association with LSM was observed. Moreover, higher serum selenium concentrations were positively associated with an increased prevalence of MASLD and a significant reduction in the risk of liver fibrosis.

## Supporting information

S1 Table Characteristics of participants. (DOCX)

S2 Table Logistic regression analysis of serum selenium and MASLD, liver fibrosis diagnosed by HIS/FIB-4. (DOCX)

S3 Table Characteristics of participants based on propensity score matching. (DOCX)

S4 Table Logistic regression analysis of serum selenium and MASLD, liver fibrosis after propensity score matching. (DOCX)

S1 File The data from NHANES used in our analysis. (XLSX)
