# Supplementary material for: Association of serum selenium with MASLD and liver fibrosis: A cross-sectional study
Source: PLoS One. 2024 Dec 31;19(12):e0314780. doi: 10.1371/journal.pone.0314780 (PMC11687858; doi:10.1371/journal.pone.0314780)
Supplement: S1 Table — (DOCX) [file pone.0314780.s001.docx]

S1 Table. Characteristics of participants

|  |  | Quartile1(<168.38ug/L) n=1679 | Quartile2(168.38-183.33ug/L) n=1678 | Quartile3(183.33-200.07ug/L) n=1674 | Quartile4(≥200.07ug/L) n=1684 | P-value | |
| --- | --- | --- | --- | --- | --- | --- | --- |
| Characteristics | |  |  |  |  |  |  |
| Age, years | | 44.57(1.16) | 43.51(0.94) | 43.80(1.02) | 45.63(0.92) | 0.035 | |
| Gender (n,%) | | | | | | <0.001 | |
| Male | | 732(43.60%) | 793(47.26%) | 840(50.18%) | 894(53.09%) |  | |
| Female | | 947(56.40%) | 885(52.74%) | 834(49.82%) | 790(46.91%) |  | |
| Race (n,%) | | | | | | <0.001 | |
| Mexican American | | 161(9.59%) | 190(11.32%) | 248(14.82%) | 224(13.30%) |  | |
| Other Hispanic | | 187(11.14%) | 181(10.79%) | 155(9.26%) | 155(9.20%) |  | |
| Non-Hispanic White | | 543(32.34%) | 592(35.28%) | 620(37.04%) | 614(36.46%) |  | |
| Non-Hispanic black | | 534(31.80%) | 463(27.59%) | 363(21.68%) | 343(20.37%) |  | |
| Other | | 254(15.13%) | 252(15.02%) | 288(17.20%) | 348(20.67%) |  | |
| Education level (n,%) | | | | | | 0.162 | |
| Less than high school | | 342(19.33%) | 319(19.01%) | 334(19.95%) | 307(18.23%) |  | |
| High school or equivalent | | 439(26.14%) | 419(24.97%) | 373(22.28%) | 398(23.63%) |  | |
| Above high school Above high school | | 898(54.53%) | 947(56.02%) | 967(57.77%) | 979(68.14%) |  | |
| Marital status (n,%) | | | | | | <0.001 | |
| Married/cohabitant | | 1101(65.57%) | 1129(68.28%) | 1159(69.24%) | 1189(70.60%) |  | |
| Widowed/divorced/separated | | 320(19.06%) | 297(17.70%) | 289(17.26%) | 235(16.63%) |  | |
| Never married | | 258(15.37%) | 252(15.02%) | 226(13.50%) | 215(12.77%) |  | |
| Physical activity (n, %) | | | | | | 0.783 | |
| Never | | 470(27.99%) | 443(26.40%) | 407(24.31%) | 413(24.54%) |  | |
| Insufficient | | 258(15.36%) | 259(15.43%) | 267(15.94%) | 278(16.50%) |  | |
| Constant | | 951(56.63%) | 976(58.17%) | 1000(59.75%) | 993(58.96%) |  | |
| Drinking status (n,%) | | | | | | <0.001 | |
| Non | | 901(53.66%) | 806(48.03%) | 775(46.30%) | 799(47.45%) |  | |
| Low to moderate | | 778(46.34%) | 872(51.97%) | 899(53.70%) | 885(52.55%) |  | |
| Cotinine status (n,%) | | | | | | <0.001 | |
| Low | | 569(33.88%) | 648(37.01%) | 805(40.98%) | 254(40.80%) |  | |
| Moderate | | 760(45.27%) | 691(41.18%) | 697(41.64%) | 743(44.12%) |  | |
| High | | 350(20.85%) | 366(21.81%) | 291(17.38%) | 254(15.08%) |  | |
| Diabetes (n,%) | | | | | | 0.001 | |
| Yes | | 262(15.60%) | 275(16.39%) | 275(16.43%) | 342(20.31%) |  | |
| No | | 1417(84.40%) | 1403(82.21%) | 1399(84.13%) | 1342(79.69%) |  | |
| Hypertension (n,%) | | | | | | <0.001 | |
| Yes | | 742(44.19%) | 754(44.93%) | 758(45.28%) | 885(52.55%) |  | |
| No | | 937(55.81%) | 924(55.07%) | 916(54.72%) | 799(47.45%) |  | |
| BMI,kg/m^2^ (n,%) | | | | | | 0.167 | |
| <28 | | 859(51.16%) | 885(52.74%) | 872(52.09%) | 827(49.11%) |  | |
| ≥28 | | 820(48.84%) | 793(47.26%) | 802(47.91%) | 857(50.89%) |  | |
| WC(cm) | | 96.68(0.90) | 97.17(1.05) | 97.09(0.85) | 98.54(0.87) | <0.001 | |
| TG (mmol/L) | | 1.01(0.04) | 1.09(0.04) | 1.10(0.03) | 1.30(0.04) | <0.001 | |
| TC (mmol/L) | | 4.53(0.06) | 4.68(0.06) | 4.65(0.05) | 4.94(0.06) | <0.001 | |
| LDL (mmol/L) | | 2.67(0.05) | 2.79(0.05) | 2.74(0.05) | 2.96(0.05) | <0.001 | |
| HDL (mmol/L) | | 1.39(0.02) | 1.40(0.02) | 1.40(0.02) | 1.38(0.03) | 0.450 | |
| FPG(mmol/L) | | 6.09(0.15) | 5.79(0.04) | 5.93(0.07) | 6.13(0.07) | <0.001 | |
| Hb1Ac(%) | | 5.68(0.06) | 5.56(0.03) | 5.58(0.03) | 5.64(0.04) | <0.001 | |
| CAP(dB/m) | | 253.09(3.31) | 252.69(3.47) | 257.70(3.29) | 266.49(2.88) | <0.001 | |
| LSM(kPa) | | 5.54(0.18) | 5.70(0.27) | 5.31(0.13) | 5.51(0.21) | 0.342 | |
| MASLD (n,%) | | | | | | <0.001 | |
| Yes | | 774(46.10%) | 786(46.84%) | 909(54.30%) | 956(56.77%) |  |  |
| No | | 905(53.90%) | 892(53.16%) | 765(45.70%) | 728(43.23%) |  | |
| Liver fibosis (n,%) | | | | | | 0.015 | |
| Yes | | 246(14.65%) | 232(13.83%) | 192(11.47%) | 251(14.90%) |  |  |
| No | | 1433(85.35%) | 1446(86.17%) | 1482(88.53%) | 1433(85.10%) |  |  |
