# Supplementary material for: Association of serum selenium with MASLD and liver fibrosis: A cross-sectional study
Source: PLoS One. 2024 Dec 31;19(12):e0314780. doi: 10.1371/journal.pone.0314780 (PMC11687858; doi:10.1371/journal.pone.0314780)
Supplement: S2 Table — (DOCX) [file pone.0314780.s002.docx]

S2 Table. Logistic regression analysis of serum selenium and MASLD, Liver fibrosis diagnosed by HIS/FIB-4

|  |  |  | Q1 | Q2 | Q3 | Q4 |
| --- | --- | --- | --- | --- | --- | --- |
| MASLD | model1 | OR (95%CI) | ref | 0.902(0.733,1.109) | 1.179(0.957,1.454) | 1.189(0.962,1.469) |
|  |  | P trend | ref | 0.327 | 0.123 | 0.110 |
|  | model2 | OR (95%CI) | ref | 0.828(0.624,1.099) | 1.219(0.936,1.589) | 1.090(0.808,1.471) |
|  |  | P trend | ref | 0.191 | 0.142 | 0.572 |
|  | model3 | OR (95%CI) | ref | 0.811(0.608,1.081) | 1.218(0.926,1.601) | 1.042(0.763,1.424) |
|  |  | P trend | ref | 0.154 | 0.159 | 0.796 |
| Liver fibrosis | model1 | OR (95%CI) | ref | 0.516(0.302,0.880) | 0.585(0.332,1.029) | 0.471(0.262,0.847) |
|  |  | P trend | ref | 0.015 | 0.063 | 0.012 |
|  | model2 | OR (95%CI) | ref | 0.558(0.321,0.970) | 0.669(0.377,1.187) | 0.436(0.239,0.797) |
|  |  | P trend | ref | 0.039 | 0.170 | 0.007 |
|  | model3 | OR (95%CI) | ref | 0.567(0.322,1.000) | 0.694(0.390,1.235) | 0.441(0.243,0.801) |
|  |  | P trend | ref | 0.050 | 0.214 | 0.007 |
