# Supplementary material for: Association of serum selenium with MASLD and liver fibrosis: A cross-sectional study
Source: PLoS One. 2024 Dec 31;19(12):e0314780. doi: 10.1371/journal.pone.0314780 (PMC11687858; doi:10.1371/journal.pone.0314780)
Supplement: S3 Table — (DOCX) [file pone.0314780.s003.docx]

S3 Table. Characteristics of participants based on propensity score matching

|  |  | Non-MASLD(n=3290) | MASLD(n=3290) | P-value | Non-Liver fibrosis(n=921) | Liver fibrosis(n=921) | P-value |
| --- | --- | --- | --- | --- | --- | --- | --- |
| Age (year) | | 38.56(0.50) | 51.52(0.41) | <0.001 | 51.74(0.93) | 50.44(0.88) | 0.672 |
| Gender(n,%) | | | | <0.001 |  |  | 0.707 |
| Male | | 1519(46.17) | 1659(50.43) |  | 518(56.24) | 526(57.11) |  |
| Female | | 1771(53.83) | 1631(49.57) |  | 403(43.76) | 395(42.89) |  |
| Race(n,%) | | | | <0.001 |  |  | 0.777 |
| Mexican American | | 331(10.06) | 467(14.19) |  | 117(12.70) | 117(12.70) |  |
| Other Hispanic | | 315(9.58) | 347(10.55) |  | 103(11.18) | 92(9.99) |  |
| Non-Hispanic white | | 1112(33.80) | 1218(37.02) |  | 313(33.98) | 337(36.59) |  |
| Non-Hispanic black | | 948(28.81) | 737(22.40) |  | 280(30.40) | 268(29.10) |  |
| Other | | 584(17.75) | 521(15.84) |  | 108(11.74) | 107(11.62) |  |
| Education level(n,%) | | | | <0.001 |  |  | 0.902 |
| Less than high school | | 330(10.03) | 570(17.32) |  | 147(15.96) | 154(16.72) |  |
| High school or equivalent | | 515(15.65) | 700(21.28) |  | 211(22.91) | 211(22.91) |  |
| Above high school | | 2445(74.32) | 2020(61.40) |  | 563(61.13) | 556(60.37) |  |
| Marital status (n, %) | | | | <0.001 |  |  | 0.756 |
| Married/cohabitant | | 1229(37.36) | 1945(59.12) |  | 466(50.60) | 482(52.33) |  |
| Widowed/divorced/separated | | 474(14.41) | 711(21.61) |  | 218(23.67) | 211(22.91) |  |
| Never married | | 1587(48.23) | 634(19.27) |  | 237(25.73) | 228(24.76) |  |
| Poverty income ratio (n, %) | | | | 0.235 |  |  | 0.375 |
| <1.30 | | 857(26.05) | 772(23.47) |  | 234(25.41) | 234(25.41) |  |
| 1.30-3.50 | | 1076(32.71) | 1129(34.32) |  | 316(34.31) | 336(36.48) |  |
| >3.50 | | 1357(41.24) | 1389(42.21) |  | 371(40.28) | 342(38.11) |  |
| Drinking status (n, %) | | | | <0.001 |  |  | 0.815 |
| Non | | 1775(53.95) | 1401(42.58) |  | 423(45.93) | 418(45.39) |  |
| Low to moderate | | 1515(46.05) | 1889(57.42) |  | 498(54.07) | 503(54.61) |  |
| Smoking habits (n, %) | | | | 0.278 |  |  | 0.691 |
| Never | | 1211(36.80) | 1301 (39.54) |  | 354(38.43) | 337 (36.59) |  |
| Former | | 1409(31.60) | 1412(30.23) |  | 404(43.87) | 412(44.73) |  |
| Current | | 1409(31.60) | 1412(30.23) |  | 163(17.70) | 1412(18.68) |  |
| Physical activity (n, %) | | | | <0.001 |  |  | 0.251 |
| Never | | 1391(42.28) | 1030(31.31) |  | 359(38.98) | 326(35.40) |  |
| Insufficient | | 278(8.45) | 409(12.43) |  | 114(12.38) | 114(12.38) |  |
| Constant | | 1621(49.27) | 1851(56.26) |  | 448(48.64) | 481(52.22) |  |
| Diabetes(n, %) | | | | <0.001 |  |  | 1.000 |
| Yes | | 215(6.53) | 939(28.54) |  | 341(37.02) | 341(37.02) |  |
| No | | 3075(93.47) | 2351(71.46) |  | 580(62.98) | 580(62.98) |  |
| Hypertension(n, %) | | | | <0.001 |  |  | 0.920 |
| Yes | | 1029(31.28) | 2107(64.04) |  | 629(68.30) | 627(68.08) |  |
| No | | 2261(68.72) | 1183(35.96) |  | 292(31.70) | 294(31.92) |  |
| BMI (kg/m2) | | | | <0.001 |  |  | 0.958 |
| <28 | | 2531(76.08) | 828(75.47) |  | 251(27.25) | 252(27.36) |  |
| ≥28 | | 759(23.92) | 2462(24.53) |  | 670(72.75) | 669(72.64) |  |
