# Supplementary material for: Association of serum selenium with MASLD and liver fibrosis: A cross-sectional study
Source: PLoS One. 2024 Dec 31;19(12):e0314780. doi: 10.1371/journal.pone.0314780 (PMC11687858; doi:10.1371/journal.pone.0314780)
Supplement: S4 Table — (DOCX) [file pone.0314780.s004.docx]

S4 Table. Logistic regression analysis of serum selenium and MASLD, Liver Fibrosis after propensity score matching

|  |  |  | Q1 | Q2 | Q3 | Q4 |
| --- | --- | --- | --- | --- | --- | --- |
| MASLD | model1 | OR (95%CI) | ref | 0.992(0.804,1.224) | 1.408(1.139,1.742) | 1.624(1.309,2.014) |
|  |  | P trend | ref | 0.938 | 0.002 | 0.001 |
|  | model2 | OR (95%CI) | ref | 0.990(0.760,1.290) | 1.510(1.168,1.954) | 1.570(1.189,2.073) |
|  |  | P trend | ref | 0.767 | 0.002 | 0.001 |
|  | model3 | OR (95%CI) | ref | 0.974(0.750,1.266) | 1.511(1.167,1.957) | 1.512(1.142,2.002) |
|  |  | P trend | ref | 0.844 | 0.002 | 0.004 |
| Liver fibrosis | model1 | OR (95%CI) | ref | 0.595(0.405,0.876) | 0.604(0.402,0.907) | 0.539(0.367,0.792) |
|  |  | P trend | ref | 0.009 | 0.015 | 0.002 |
|  | model2 | OR (95%CI) | ref | 0.578(0.388,0.861) | 0.575(0.383,0.864) | 0.520(0.352,0.770) |
|  |  | P trend | ref | 0.007 | 0.008 | 0.001 |
|  | model3 | OR (95%CI) | ref | 0.570(0.383,0.849) | 0.566(0.377,0.850) | 0.514(0.346,0.763) |
|  |  | P trend | ref | 0.006 | 0.006 | 0.001 |
